# Clinical outcome and concomitant injuries in operatively treated fractures of the lateral process of the talus

**DOI:** 10.1186/s12891-019-2603-3

**Published:** 2019-05-15

**Authors:** Hubert Hörterer, Sebastian Felix Baumbach, Stefan Lemperle, Sebastian Altenberger, Oliver Gottschalk, Alexander Tobias Mehlhorn, Anke Röser, Markus Walther

**Affiliations:** 1 Center for Foot and Ankle Surgery, Schön Klinik München Harlaching, München, Germany; 2 Department of General, Trauma, and Reconstructive Surgery, University Hospital, LMU Munich, Munich, Germany; 3 Center for Foot and Ankle Surgery, Schön Klinik Bad Aibling Harthausen, Bad Aibling, Germany; 4 Department of Trauma and Orthopedic Surgery, University Hospital Freiburg, Freiburg, Germany; 5 Department of Orthopedics and Orthopedic Surgery, Julius-Maximilians-University, Würzburg, Germany

**Keywords:** Fracture, snowboarder’s ankle, snowboarder’s fracture, Lateral process of the talus

## Abstract

**Background:**

The aim of this study was to review the patient rated outcome (PROM) of surgically treated fractures to the lateral process of the talus (LPTF) and identify factors influencing the outcome.

**Methods:**

Retrospective study with a current follow-up. Eligible were all patients treated surgically for a LPTF (*n* = 23) with a minimum follow-up of one year. Demographics, medical history, trauma mechanism, fracture characteristics, concomitant injuries, treatment details, complications, return to work and sports were assessed retrospectively. The current follow-up included the VAS FA, Karlsson Score, and SF-12. The primary outcome was the VAS FA. Secondary aim was the identification of parameters influencing the PROMs.

**Results:**

22 patients (96% follow-up) with a mean age of 32 ± 9 (18 to 49) years were included. 73% suffered a Hawkins Type 1, 23% a Type 2, and one patient a Type 3 fracture. 82% suffered concomitant injuries. 9% suffered minor surgical side infections, 50% developed symptomatic subtalar osteoarthritis. At final follow-up (44 ± 2 (12 to 97) months), the mean VAS FA Overall was 77 ± 21 (20 to 100), the Karlsson Score 72 ± 21 (34 to 97), and for the SF 12 the PCS 53 ± 8 (36 to 64) and the MCS 53 ± 7 (32 to 63). 50% of patients returned to their previous level of sports. Hawkins Type 1 fractures resulted in better VAS FA Overall score than Type 2 fractures. Posttraumatic subtalar osteoarthritis was the independent factor associated to a poor patient rated outcome (VAS FA, Karlsson Score).

**Conclusion:**

After a follow-up of over 3.5 years, surgically treated LPTF resulted in only moderate results. 50% suffered posttraumatic symptomatic subtalar osteoarthritis, which was the primary independent parameter for a poor outcome following LPTF.

**Level of evidence:**

Level III.

**Electronic supplementary material:**

The online version of this article (10.1186/s12891-019-2603-3) contains supplementary material, which is available to authorized users.

## Background

The talus is the second largest tarsal bone with a broad-based triangular lateral process on its lateral side, i.e. the lateral process of the talus (LPT). It articulates with the fibula superiorly and builds the anterior-lateral portion of the posterior subtalar joint [1].

Overall, LPT fractures (LPTF) are rare [2] but have been stated to account for up to 20% of all talar fractures [3]. They are traditionally attributed to a high energy trauma mechanism [4–6]. More recent studies have shown a 17-fold fracture risk increase in snowboarders. Consequently, LPTF are also referred to as “Snowboarders Ankle” or “Snowboarders Fracture” [7–10]. The actual trauma mechanism is a matter of debate. Based on the data available, forced dorsiflexion, inversion, eversion, and potentially external rotation have been postulated [11–13].

Diagnosis of LPTF is challenging, as almost 50% of fractures are missed on plain radiographs [6, 14]. Consequently, computed tomography (CT) must be performed in case of suspicion [14, 15]. Various foot and ankle classification systems, such as the AO-classification or ICI classification, do included LPTF. Still, Hawkins [6] as well as McCrory and Bladin [16] are the only two classifications, specifically focusing on LPTF. The Hawkins classification, published in 1965, thereby is still the most commonly used. It classifies LPTF as simple fractures (Type I), comminuted fractures (Type II), or chip fracture (Type III) [6]. The McCrory and Bladin classification [16] describes similar fracture patterns just in a different order. Both classifications are based on plain radiographs and therefore might underestimate the complexity of these fractures. Studies including CT or MRI imaging reported concomitant injuries in 46 to 88% of patients suffering a LPTF [15, 17]. Sadly, up to date we are missing a comprehensive classification for LPTF including concomitant injuries. In order to present the data available in literature more uniformly, all fractures were re-classified per the Hawkins classification [6].

The best treatment for LPTF remains a matter of debate as these fractures are rare, the literature available is limited, and concomitant injuries are frequent. The vast majority of LPTF studies are descriptive, retrospective case series based on plain radiographs missing a standardized treatment concept [4–6, 10, 17, 18]. Only two studies validated a predefined treatment regimen [7, 15]. Valderrabano et al. [7] treated fractures with a displacement of more than 2 mm surgically. They presented the outcome of six conservatively (Type I: 2, Type II: 1, Type III: 3) and 14 operatively (Type I: 14) treated patients. In this cohort, operative treatment resulted in superior AOFAS scores compared to the conservative treatment (AOFAS: 97 ± 1) vs. 85 ± 5). Von Knoch et al. [15] recommended surgery in case of displacement of more than 1 mm. Seven patients were treated conservatively (Type I: 4, Type II: 2, Type III: 1), 16 operatively (Type I: 11, Type II: 2). In contrast to Valderrabano et al. [7], this study reported superior results for the conservative treatment (AOFAS: 98 (87–100) vs. 93 (82–100)). Not only do those two studies report contradictory results, but they are also limited because of their group sizes and the chosen outcome measure (AOFAS). Although the AOFAS is one of the most frequently applied scores, it has been questioned for its validity, responsiveness and applicability [19–22]. Consequently, we are missing an evidence-based treatment algorithm for LPTF.

At the authors’ orthopedic reference center, patients presenting with a displaced Hawkins Type I or Type II fracture as well as patients suffering relevant concomitant injuries, such as subtalar osteochondral lesions, loose bodies, or peroneal tendon dislocation, are treated surgically. Undisplaced fractures (< 2 mm) and Hawkins Type III fractures without relevant concomitant injuries are treated conservatively. In case of conservative treatment, the further treatment is conducted by the referring physician. Injuries necessitating operative treatment remain in the authors’ reference center.

The way outcome in orthopedic trauma is being assessed is changing, shifting to patient focused outcome measures such as quality of life (QOL) and patient-reported outcome measures (PROMs) [23–25]. From 1985 to 2015, the number of studies on injured patients including PROMs increased almost 30-fold [23]. PROMs, per the recommendations of the COSMIN group, should be valid, reliable, and responsive [26–28]. Today, various PROMs have been published, but we are still missing a universally applied gold standard [21]. In this study, we applied the VAS-FA, Karlsson Score and SF-12, which were the standard PROMs used in our department at that time.

The primary aim of this study was to review the PROM of all surgically treatment patients suffering a LPTF with a follow-up of at least one year. The secondary aim was to identify factors affecting the PROM.

## Methods

### Study design

The study design is a retrospective cohort study with a current follow-up of at least one-year. The study was approved by the local ethic committee (Julius-Maximilians University of Wuerzburg; Approval Nr. 94/17).

### Treatment protocol

At our center, patients suffering a Hawkins Type I or Type II fracture as well as patients presenting with relevant concomitant injuries (for example subtalar osteochondral lesions/loose bodies or peroneal tendon dislocation), are treated surgically. All fractures were assessed on CT imaging and classified according to Hawkins. MRI was conducted per the preference of the treating orthopedic consultant. Hawkins Type 1 fractures are addressed by open reduction and internal screw fixation. For Type 2 and 3 fractures, the fragments are resected. Moreover, all concomitant injuries are addressed. The postoperative protocol is restrictive. Patients are advised to wear a walker for 8 weeks with partial-weightbearing for 6 weeks. Physiotherapy is initiated immediately postoperative. The postoperative regime might be adapted per the concomitant injuries.

### Patient selection

**Fig. 1:**
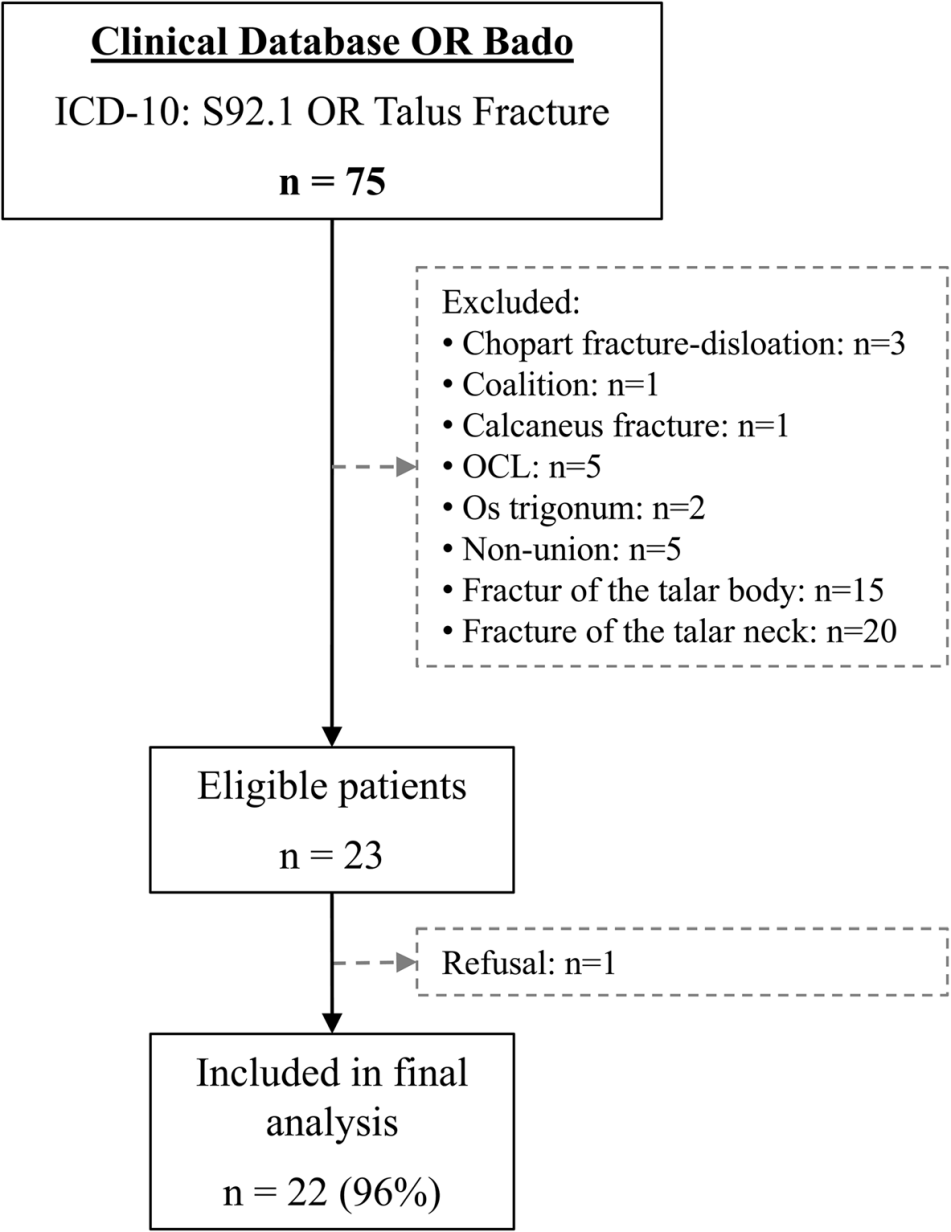
Patient selection flow-chart

The patient identification was based on the department’s clinical database, which was searched for the ICD-10 S92.1 from 01/2009 to 09/2016. The inclusion criteria were age > 18 years, acute fracture of the LPT, initial CT imaging, and a current follow-up of at least one year. Exclusion criteria were preexisting conditions or concomitant fractures, which possibly could have influenced the functional outcome, as well as the inability to give informed consent. The overall patient selection is illustrated in Fig. 1.

### Data generation

The following data were assessed retrospectively based on the digital patient records: General demographics (age, height, weight, BMI), medical history, trauma mechanism, fracture details, treatment details, return to work and sports, as well as any complications. Fracture details were assessment on CT scans and MRI where available. Fractures were classified according to Hawkins [6] and subclassified for initial displacement (< / > 2 mm) and number of fragments (single- or multi-fragmentary). Any concomitant injury was recorded. Based on the documentation available, complications including surgical side infections, revision surgeries and secondary symptomatic osteoarthritis were assessed. Symptomatic osteoarthritis was appraised based on clinical examination and radiographs.

Patients were invited to complete the following PROMs as a current follow-up: Visual-Analogue-Scale Foot and Ankle (VAS FA), Karlsson Score, and a general quality of life score (SF-12) [29–31]. The VAS FA scores between 0 and 100 for each of its four domains (overall, pain, function, other), with 100 resembling no foot or ankle pain [29]. The Karlsson Score has been designed to assess ankle joint function following lateral ankle ligament repair with a scoring range from 0 to 100, with 100 points resembling preinjury levels of activity [30]. The SF-12 is one of the most common quality of live scores. It scores in 8 subdomains which again are subsummarized to a physical (PCS) and mental (MCS) domain. Next to the PCS and MCS, the physical function (PF) and the bodily pain (BP) subscales were chosen for further analysis. Scores of 50 equal those of a health reference population [31].

### Outcome parameters

The VAS FA was chosen as primary outcome parameter as it was the most comprehensive and best-validated PROM assessed herein. Secondary outcome parameters were return to work and sports, Karlsson Score, and SF-12. The influence of the demographics (age, sex, BMI), trauma mechanism (low- / high velocity), fracture characteristics (Hawkins classification, comminution), concomitant injuries and complications on the assessed PROMs was evaluated. Finally, the individual patterns of concomitant injuries were analyzed to hypothesis a possible trauma mechanism.

### Statistics

Due to the limited incidence of LPTF and the study design, no sample size calculation was conducted. If not stated differently, values are given as mean ± standard deviation (range). Differences were assessed using the Chi-Square test and correlations calculated using the Pearson correlation coefficient. Due to multiple testing, an alpha-level correction (Bonferroni) was performed for the secondary outcome parameters, setting the level of significance to *p* ≤ 0.005. All statistics were performed using SPSS Vs. 22 (IBM Corp, Armonk, NY).

## Results

Overall, 75 patients were identified from the database, 23 were eligible and 22 (96%) includ.ed in the final analysis (Fig. 1). Their mean age was 32 ± 9 (18 to 49) years, 73% were male with a mean BMI of 23 ± 3 (19 to 28) kg/m^2^. No relevant preexisting medical conditions, including smoking or diabetes, were reported.

### Trauma & Fracture characteristics

The left side was affected in 23% of the patients. A low velocity trauma mechanism (distorsion) was reported in 23%. High velocity trauma mechanisms included a fall during rock climbing (32%), or following a jump during snowboarding (14%). Each fracture was assessed by CT. An MRI was available in 46% of the patients. Table 1 summarized the assessed fracture characteristics. Neither displacement (*p* = 0.521), nor comminution (*p* = 0.060), age (*p* = 0.189.), gender (*p* = 0.160), or BMI (*p* = 0.754) differed significantly between the Hawkins classification (Type 1 and 2).

**Table 1:** Fracture characteristics, concomitant injuries, and treatment details per Hawkins classification

Hawkins classification	Type 1 *n*=16 (73%)	Type 2 *n*=5 (23%)	Type 3 *n*=1 (5%)
Displacement ≥2 mm	13 (81%)	5 (100%)	1
Comminuted	6* (38%)	5 (100%)	1
Concomitant injuries	13 (73%)	4 (80%)	1
Number concomitant injuries	1.7 ± 1.1 (0 to 4)	2.0 ± 1.6 (0 to 4)	3
Primary surgical procedure			
ORIF	16 (100%)	0 (0%)	0
Resection	0 (0%)	5 (100%)	1
Additional surgical procedures			
None	5 (31%)	1 (20%)	0
Resection i.a. loose bodies	7 (44%)	0	0
Subtalar microfracturing	2 (13%)	1 (20%)	1
Subtalar AMIC	2 (13%)	0	0
Reconstruction lateral ankle ligament complex	0	2 (40%)	0
Peroneal tendon repair	0	1 (20%)	0

* As assessed on CT imaging, one main fragment accompanied by smaller chip-off fragments

**Fig. 2:**
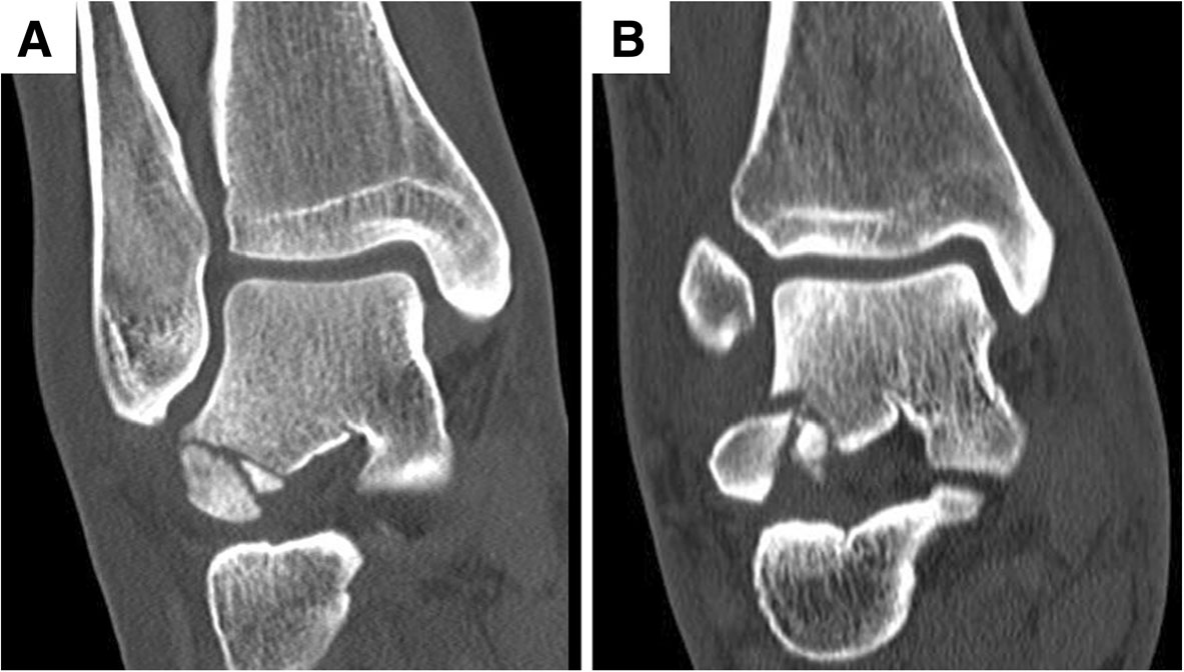
Illustration of patients classified as Hawkins Type 1 with comminution. **a** and **b**: Two patients classified as Hawkins Type 1 with comminution

Six patients were classified as Hawkins Type 1 with comminution. This discrepancy to the traditional Hawkins classification is due to the different imaging modalities. Hawkins classified all fractures based on plain radiographs [6]. In this study, all fractures were classified based on CT images. Those patients classified as Hawkins Type 1 with comminution presented one large main fragment accompanied by few small comminuted fragments (Fig. 2), which most likely would not have been visible on plain radiographs.

### Concomitant injuries

Eighty two percent of patients suffered 41 concomitant injuries, on average 1.8 ± 1.2 (0 to 4) per patient. In detail, a osteochondral lesion of the posterolateral calcaneal facet of the subtalar joint occurred in 13 (59%) patients, a lesion to the plantolateral aspect of the head of talus was present in 13 (59%; osteochondral lesion *n* = 7; BME *n* = 6), a bony avulsion of the medial talocalcaneal ligament was seen in 11 (50%) patients, a sprain of the spring ligament in 2 (9%) patients, and a lesion to the TN capsular or a luxation of the peroneal tendons in 1 (5%) patient each. Their distribution per Hawkins classification is illustrated in Table 1. Neither the occurrence (*p* = 0.888) nor the number (*p* = 0.630) of concomitant injuries differed significantly between the different fracture patterns according to Hawkins.

### Treatment details

ORIF by screw osteosynthesis was conducted for all Hawkins Type 1 fractures and resection for Type 2 and 3 fractures, one of which arthroscopically. Additional procedures were necessary in 73% of the patients (Table 1) with no significant difference per the Hawkins classification (*p* = 0.728). The duration of the in-house stay was 3.6 ± 1.5 (2 to 8) days on average. The patients conducted partial weightbearing for 7.4 ± 2.5 (2 to 10) weeks and were immobilized for 9.1 ± 3.1 (0 to 12) weeks. Neither the duration of the in-house stay (*p* = 0.881) nor of partial weightbearing (*p* = 0.052), or immobilization (*p* = 0.230) differed significantly per the Hawkins classification.

### Complications and secondary osteoarthritis

Only two patients (9%) suffered minor complications, all of which were surgical side infections resolving by conservative measures. Six (27%) patients underwent hardware removal, arthrolysis and partial synovectomy due to hardware irritation and subtalar osteoarthritis. In total, 12 (55%) patients presented in the course of their follow-up out-patient visits with symptomatic subtalar osteoarthritis, with no significant differences (*p* = 0.189) between Hawkins Type 1 (50%) and 2 (60%) fractures.

### Patient rated outcome

Patients returned to work after 63 ± 47 (7 to 200) days and to sports after 8 ± 5 (3 to 24) months after the initial surgery. Again, the Hawkins classification had no significant influence on the time to return to work (*p* = 0.813) or sports (*p* = 0.368).

**Fig. 3:**
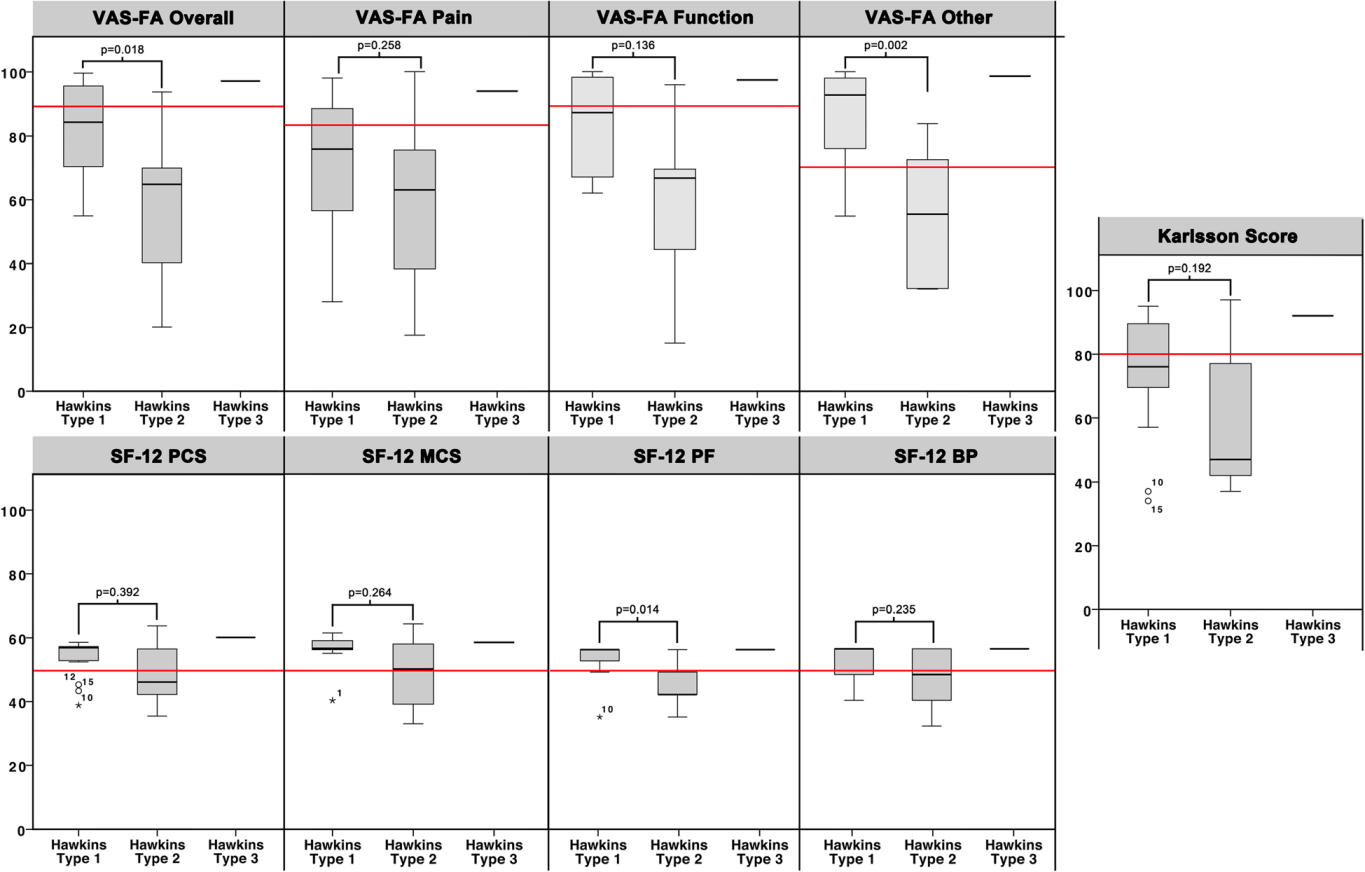
Patient rated outcome measures per the Hawkins classification. Red line: Lower-bound reference value of published norm values for a healthy population (VAS-FA: (Overall: 86; Pain: 82; Function: 87; Other: 68) [29]; Karlsson Score: 80 [30]; SF-12: 50)

The mean final follow-up was 44 ± 2 (12 to 97) months. At that time, the mean VAS FA overall was 77 ± 21 (20 to 100), the Karlsson Score 72 ± 21 (34 to 97), and for the SF 12 the PCS 53 ± 8 (36 to 64), the MCS 53 ± 7 (32 to 63), the PF 52 ± 8 (34 to 57), and the BP 53 ± 8 (31 to 58). The PROMs’ distribution per the Hawkins classification is illustrated in Fig. 3. Only 50% of patients have returned to their previous level (≥90%) of sports.

### Factors influencing the PROM

In order to identify factors affecting the patient rated outcome, the influence of age, BMI, gender, trauma mechanism (low- / high velocity), fracture characteristics (Hawkins classification, comminution), concomitant injuries (binary and number), complications (none, subtalar osteoarthritis), and revision surgery on the assessed PROMs (VAS FA, Karlsson Score, SF-12) was evaluated. The detailed statistics are presented in Additional file 1. There was a significant difference (*p* = 0.002) for the VAS FA Other subscale between Hawkins Type 1 (86 ± 15) and Type 2 (55 ± 23) fractures. Moreover, patients suffering from posttraumatic subtalar osteoarthritis had significant worse scores for the VAS FA Overall (94 ± 6 vs. 65 ± 21; p = 0.002), −Pain (86 ± 10 vs. 57 ± 22; p = 0.002), −Function (95 ± 6 vs. 67 ± 22; p = 0.002), −Other (97 ± 4 vs. 69 ± 23; *p* = 0.001), and the Karlsson Score (85 ± 11 vs. 60 ± 20; *p* = 0.003).

## Discussion

This study presents the largest cohort on surgically treated LPT fractures. Hawkins Type 1 fractures were treated by open reduction and screw fixation, Type 2 and 3 fractures by resection of the fracture fragments. Furthermore, all concomitant injuries were addressed. Despite this progressive treatment approach, after more than three years after the surgery, the patient rated outcome revealed only moderate to good scores. Twelve (55%) patients suffered from posttraumatic symptomatic subtalar osteoarthritis. Although Hawkins Type 1 fractures resulted in better VAS FA Overall scores than Type 2 fractures, the only independent factor associated to an impaired patient rated outcome was posttraumatic symptomatic subtalar osteoarthritis.

The authors are only aware of eight case series (> 6 patients) reporting on the outcome of LPTFs [4–7, 10, 17, 18]. Out of those, only two studies applied a standardized treatment algorithm and evaluated the surgical outcome using PROMs [7, 15]. Valderrabano et al. reported excellent results for 14 operatively treated Hawkins Type I fractures. Their mean AOFAS score was 97 (1) and a VAS of 0.2 (0.6) points [7]. However, no data was reported for operatively treated Type II fractures. Von Knoch et al. reported good to moderate AOFAS scores of 93 (82 to 100) points for operatively treated displaced Type I (*n* = 11) and Type II (*n* = 5) fractures [15]. Still, various studies have questioned the validity of the AOFAS [19, 20, 22]. As outlined in the introduction, the fractures included in the study by Valderrabano et al. [7] and von Knoch et al. [15] were transformed to the Hawkins classification to increase the comparability.

The herein presented 22 operatively treated LPTFs resulted in only moderate to good PROMs. The VAS FA and Karlsson Score revealed residual impairment. The VAS FA Overall Score (77 ± 21 (20 to 100)) was lower than the published reference values for healthy individuals (86 to 100) but comparable to patients with an isolated hallux valgus (45 to 83) [32]. Similar results were found for the Karlsson Score (72 ± 21 (34 to 97)) with scores above 80 points representing good to very good results [30]. The patients’ quality of life (SF-12: PCS 53 ± 8 (36 to 64), MCS 53 ± 7 (32 to 63)) was in the range of a healthy population. Overall, operatively treated displaced Type I or II fractures result in good to moderate results.

The observed residual impairment was also reflected in the return to sports rate. Only half of the patients reached a sport activity level ≥ 90% after on average 8.2 ± 4.9 (3 to 24) months. A surprisingly high rate (100%) of return to sports in operatively treated patients was found by Valderrabano et al. [7] Von Knoch et al. [15] and Klein et al. [17] reported rates of return to sports comparable to the herein presented study (63% / 59%).

The secondary aim of this study was to identify factors associated to a poor patient rated outcome. Although Hawkins Type 1 fractures resulted in superior results compared to Type 2 fractures (VAS FA Overall: *p* = 0.018 and -Other: *p* = 0.002) the strongest independent factor for impairment was posttraumatic symptomatic subtalar osteoarthritis (VAS FA and Karlsson Score). In the current study, 50% of patients suffered from symptomatic subtalar osteoarthritis, which compares well to literature with reported rates of 15 to 45% [6, 7, 10]. On the contrary, neither the occurrence nor the number of concomitant injuries affected the patient rated outcome. Only five studies have previously reported on concomitant injuries following LPTFs [6, 10, 15, 17, 18]. Von Knoch et al. reported a rate of concomitant injuries of 88% [15]. Klein et al. reported on peroneal tendon displacement in 46% of patients suffering a Type 2 LPTF [17]. Both studies did not further analyze the influence of these concomitant injuries. These figures are in the range of the herein observed rate of 82% of patients suffering concomitant injuries. This number might even underestimate the actual occurrence of concomitant injuries accompanying LPTFs, as several of these injuries are only detectable on MRI, and MRI was available for just 46% of patients in this study. On the contrary, the high number of concomitant injuries could have hindered sufficient statistical analysis.

**Fig. 4:**
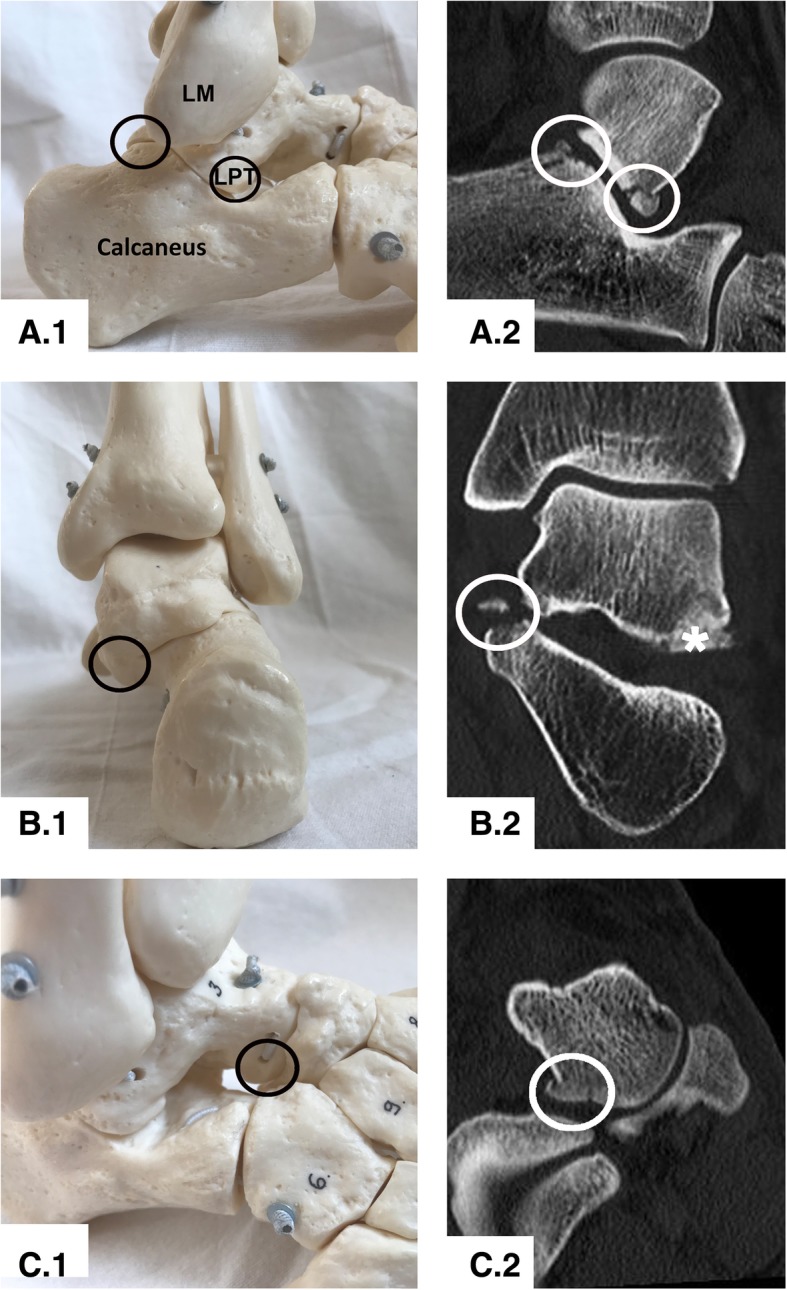
Illustration of frequent concomitant injuries. The black and white circles illustrate the lesion; 1) Illustration of the location of the concomitant injuries on a foot and ankle model; 2) Corresponding CT imaging of the presented lesion; The lesions are highlighted by circles; **a**) Lateral View of the subtalar joint with an osteochondral lesion to the posterolateral calcaneal facet and the LPTF; **b**) Dorsal view of the subtalar joint showing the bony avulsion of the medial talocalcaneal ligament and the LPTF on the CT image (*); **c**) Dorsolateral view of the Chopart joint line with a lesion to the plantolateral aspect of the talar head

The high incidence of symptomatic posttraumatic subtalar osteoarthritis and the concomitant injuries observed (osteochondral lesion to the posterolateral calcaneal facet, bony avulsion of the medial talocalcaneal ligament, lesion to the plantolateral aspect of the talar head), might have implications for the actual trauma mechanism (Fig. 4). Up to now, most authors discuss a combination of axial compression, dorsal extension, inversion and external rotation of the foot [4–6, 12, 13, 33]. Taking into consideration the above outlined combination of concomitant injuries, the mechanism of injury could also be a subluxation of the subtalar joint with external rotation and pronation (Fig. 4).

Several limitations of the herein presented study need to be discussed. First, no sample size calculation was conducted due to the observational study design and the low incidence of LPTF. Second, the study was retrospective, which again is in line with most other studies and again attributable to the low fracture incidence [4–6, 10, 15, 17, 18]. A further limitation could be the small number of patients (*n* = 22). Still, this is the largest published cohort on surgically treatment of LPTF [5–7, 10, 15]. The most pronounce limitation is a missing control group. Up to now, extensive comparative studies are missing completely. Although Valderrabano et al. and von Knoch et al. reported on the AOFAS of conservatively treated LPTF, they did not compare different treatment regimes in similar fracture patterns and reported conflicting results [7, 15]. It would be of great interest to prospectively asses the PROM of conservatively treated, displaced LPTF Type I and II according to Hawkins. Finally, the herein used PROMs are not fully validated per the COSMIN group recommendations [26–28] and the normative data available for the VAS-FA have not been validated in a foot and ankle trauma population [32]. Future studies should be even more aware of the PROMs chosen and consider scores such as the Self-reported Foot and Ankle Score (SEFAS) [21, 34], Manchester-Oxford foot questionnaire (MOXFQ) [35–37].

Despite the above outlined limitations, several strengths of this study are noteworthy. First the intermediate follow-up of more than three years. Second, a follow-up of 96% of patients. Third, the detailed fracture assessment based on CT and MRI imaging. The authors are not aware of any work that has investigated the concomitant injuries and fracture patterns in such detail. Finally, this is the first study to clearly show, that despite consequent treatment of all concomitant injuries, posttraumatic symptomatic subtalar osteoarthritis is the independent factor associated to an impaired patient rated outcome.

## Conclusion

The authors present the largest cohort on surgically treated LPT fractures. Despite consequent treatment of all concomitant injuries, the patient rated outcome revealed only moderate results. Although Hawkins Type 1 fractures resulted in a better outcome than Type 2 fractures, the major factor affecting the outcome was symptomatic posttraumatic subtalar osteoarthritis. The authors have hypothesized that this is due to a subtalar subluxation. Future randomized studies have to compare the patient rated outcome in conservative to surgically treated displaced LPTF.

## Additional file

Additional file 1: Influence of various factors on the patient rated outcome measures (PROMs). (DOCX 19 kb)

## Abbreviations

%: Percent
<: Smaller than
>: Greater than
±: Plus / minus
≤: Smaller or equal
AOFAS: American Orthopedic Foot and Ankle Score
BMI: Body mass index
CT: Computed Tomography
d: Days
Fig.: Figure
i.a: Inter alia
ICD-10: International Statistical Classification of Diseases and Related Health Problems by the World Health Organization
kg: Kilogram
LM: Lateral Malleolus
LPT: Lateral Process of Talus
LPTF: Lateral Process of Talus Fracture
m^2^: Squaremeter
MCS: Mental component summary
mm: Millimetre
MRI: Magnetic Resonance Imaging
ORIF: Open reduction internal fixation
p: *p*-value
PCS: Physical component summary
PROM: Patient rated Outcome Measure
SD: Standard deviation
SF-12: 12-item short-form of the SF-36 Health Survey
VAS-FA: Visual analog scale foot and ankle
Vs: Versus
