# Supplementary material for: Clinical outcome and concomitant injuries in operatively treated fractures of the lateral process of the talus
Source: BMC Musculoskelet Disord. 2019 May 15;20:219. doi: 10.1186/s12891-019-2603-3 (PMC6521553; doi:10.1186/s12891-019-2603-3)
Supplement: Supplementary file 1 — Influence of various factors on the patient rated outcome measures (PROMs). (DOCX 19 kb) [file 12891_2019_2603_MOESM1_ESM.docx]

| **Additional file 1:** Influence of various factors on the patient rated outcome measures (PROMs). | | | | | | | | | | | |
| --- | --- | --- | --- | --- | --- | --- | --- | --- | --- | --- | --- |
|  | | **Age** | **BMI** | **Gender** | **Trauma** | **Hawkins Classification** | **Communition** | **Concomitant Injuries** | **Number of Concomitant Injuries** | **Complications*** | **Revision surgery** |
| **VAS-FA** | **Overall** | r=0.013  p=0.954 | r=0.132  p=0.557 | p=0.009 | p=0.195 | p=0.018 | p=0.416 | p=0.744 | p=0.644 | **p=0.002** | p=0.132 |
|  | **Pain** | r=0.168  p=0.454 | r=0.135  p=0.550 | p=0.093 | p=0.249 | p=0.258 | p=0.942 | p=0.919 | p=0.684 | **p=0.002** | p=0.037 |
|  | **Function** | r=-0.047  p=0.834 | r=0.119  p=0.597 | p=0.005 | p=0.156 | p=0.136 | p=0.225 | p=0.614 | p=0.510 | **p=0.002** | p=0.182 |
|  | **Other** | r=0.013  p=0.954 | r=0.135  p=0.550 | p=0.063 | p=0.349 | **p=0.002** | p=0.558 | p=0.938 | p=0.998 | **p=0.001** | p=0.248 |
| **Karlsson Score** | | r=0.127  p=0.574 | r=0.229  p=0.306 | p=0.053 | p=0.082 | p=0.192 | p=0.785 | p=0.456 | p=0.994 | **p=0.003** | p=0.061 |
| **SF-12** | **PCS** | r=0.150  p=0.506 | r=0.047  p=0.837 | p=0.323 | p=0.021 | p=0.392 | p=0.613 | p=0.843 | p=0.752 | p=0.016 | p=0.153 |
|  | **MCS** | r=-0.312  p=0.157 | r=-0.318  p=0.150 | p=0.120 | p=0.655 | p=0.264 | p=0.129 | p=0.750 | p=0.811 | p=0.166 | p=0.228 |
|  | **PF** | r=-0.011  p=0.960 | r=0.092  p=0.685 | p=0.184 | p=0.091 | p=0.014 | p=0.881 | p=0.772 | p=0.334 | p=0.435 | p=0.627 |
|  | **BP** | r=0.105  p=0.642 | r=-0.116  p=0.607 | p=0.291 | p=0.006 | p=0.235 | p=0.481 | p=0.610 | p=0.869 | p=0.512 | p=0.687 |
| Concomitant injures: binary (yes / no); Complications: none, subtalar osteoarthritis; **bold values**: Values meeting the adapted level of significance of p<0.008; *Difference between no complications and subtalar osteorarthritis; PCS: Physical Composite Scale; MCS: Mental Composite Scale; PF: Physical Function subscale, BP: Bodily Pain subscale; | | | | | | | | | | | |
